# Effect of Increasing the Dietary Protein Content of Breakfast on Subjective Appetite, Short-Term Food Intake and Diet-Induced Thermogenesis in Children

**DOI:** 10.3390/nu12103025

**Published:** 2020-10-02

**Authors:** Nick Bellissimo, Tammy Fansabedian, Vincent C.H. Wong, Julia O. Totosy de Zepetnek, Neil R. Brett, Alexander Schwartz, Stephanie Cassin, Katherine Suitor, Dérick Rousseau

**Affiliations:** 1 School of Nutrition, Ryerson University, Toronto, ON M5B-2K3, Canada; tfansabe@ryerson.ca (T.F.); vincent.ch.w@gmail.com (V.C.H.W.); neil.brett@ryerson.ca (N.R.B.); alexander.sasha.schwartz@gmail.com (A.S.); katherine.suitor@ryerson.ca (K.S.); 2 Faculty of Kinesiology and Health Studies, University of Regina, Regina, SK S4S-0A2, Canada; julia.totosy@uregina.ca; 3 Department of Psychology, Ryerson University, Toronto, ON M5B-2K3, Canada; stephanie.cassin@ryerson.ca; 4 Department of Chemistry & Biology, Ryerson University, Toronto, ON M5B-2K3, Canada; rousseau@ryerson.ca

**Keywords:** diet-induced thermogenesis, dietary protein, satiety, glycemic response

## Abstract

Dietary protein affects energy balance by decreasing food intake (FI) and increasing energy expenditure through diet-induced thermogenesis (DIT) in adults. Our objective was to investigate the effects of increasing the dietary protein in an isocaloric breakfast on subjective appetite, FI, blood glucose, and DIT in 9–14 y children. Two randomized repeated measures designs were used. In experiment 1, 17 children (9 boys, 8 girls) consumed isocaloric meals (450 kcal) on four separate mornings containing: 7 g (control), 15 g (low protein, LP), 30 g (medium protein, MP) or 45 g (high protein, HP) of protein. Blood glucose and subjective appetite were measured at baseline and regular intervals for 4 h, and FI was measured at 4 h. In experiment 2, 9 children (6 boys, 3 girls) consumed the control or HP breakfast on two separate mornings, and both DIT and subjective appetite were determined over 5 h. In experiment 1, all dietary protein treatments suppressed subjective appetite compared to control (*p* < 0.001), and the HP breakfast suppressed FI compared with the LP breakfast and control (*p* < 0.05). In experiment 2, DIT was higher after HP than control (*p* < 0.05). In conclusion, increasing the dietary protein content of breakfast had favorable effects on satiety, FI, and DIT in children.

## 1. Introduction

The increasing proportion of children in Western countries categorized as overweight or obese has led to an interest in the role of a high protein diet to support healthy body weights [1]. Yet, there is a lack of effective dietary tools available for body weight maintenance in children, which highlights the need to identify potential preventative dietary strategies. It is well established that macronutrient composition plays a role in satiety and energy intake. A hierarchy exists where protein is the most satiating macronutrient compared to carbohydrates and fats, and has been shown to regulate energy intake in adults [2,3]. While the benefits of dietary protein intake on body weight maintenance and energy balance have been reported in both short- and long-term studies in adults, there is limited existing literature in children [4,5]. A recent study in participants aged 2–22 y reported that higher protein intakes in childhood were related to healthier body mass index (BMI) trajectories in young adulthood [6]. Similarly, a large, multicenter European study previously demonstrated that a small increase in protein intake combined with a small reduction in glycemic index can limit weight gain following weight loss among children aged 5–18 y [7]. In particular, previous research has associated eating breakfast with greater energy intake control and weight maintenance in children compared to breakfast skipping [8]. Therefore, examining the effects of breakfast composition, specifically protein content, on food intake and appetite regulation is essential in order to inform future nutrition recommendations for children.

Dietary protein intake may regulate energy balance by decreasing food intake (FI), increasing satiety hormones [3,9], improving glucose regulation [10,11] and increasing energy expenditure through diet-induced thermogenesis (DIT) [12]. Diet-induced thermogenesis is estimated to account for approximately 10% of daily energy expenditure in humans and is comprised of the energetic costs of postprandial processes including food breakdown, enzyme synthesis, peristalsis, and nutrient uptake and assimilation [13]. A body of literature currently exists in adults suggesting protein is not only the most thermogenic macronutrient, but also the most satiating, suggesting a potential relationship between satiety and DIT [2]. While DIT is associated with subjective satiety in both healthy weight and overweight adults [12], this relationship is still relatively unexplored in children and adolescents. In adults, dietary protein increases DIT more than carbohydrates and fat [14,15,16] due to the high-energy costs associated with protein synthesis [17] and changes in substrate utilization that favors fat oxidation [18].

Previous work in adults has shown that ~30 g of protein at a meal was needed to maximize satiety and suppress FI [19,20]. Some of these functional benefits have also been demonstrated in children, as we have demonstrated that 50 g of whey protein suppresses FI more than glucose [21]. In children [22] and adolescents [23,24], protein intakes of ≥30 g at mealtime have been shown to suppress FI compared to lower protein quantities. Protein metabolism and energy expenditure are dependent on the protein source as a function of amino acid oxidation and the high energy costs of excess protein metabolism [2]. Indeed, consuming an egg-based breakfast significantly reduced short-term energy intake in children [22]. Yet, most research has focused on dairy proteins [18,25,26], and few studies have investigated potential mechanisms of short-term FI suppression and satiety following varying non-dairy protein consumption in children.

The present study investigated the effects of dietary protein included at breakfast on DIT and subjective appetite in 9–14 y children. Our objectives were two-fold: (1) To assess the effects of increasing the dietary protein portion of an isocaloric meal on subjective appetite, glycemic response, and short-term FI (experiment 1) and, (2) to determine the effect of a high protein meal on DIT in children (experiment 2). Based on our current understanding, we hypothesized that a breakfast meal higher in protein content would increase subjective satiety and DIT to a greater extent than a breakfast meal with lower protein content. Furthermore, a breakfast meal with higher protein content would demonstrate lower carbohydrate oxidation than a breakfast meal lower in protein.

## 2. Materials and Methods

### 2.1. Participants

Seventeen boys and girls 9–14 y were recruited for both experiments through advertisement postings on an online classifieds’ website, school and community bulletin boards, and by word-of-mouth. Average baseline participant characteristics are described in Table 1. Seventeen children (*n* = 9 boys, *n* = 8 girls) participated in experiment 1, and a subset of ten individuals (*n* = 7 boys, *n* = 3 girls) participated in experiment 2. Respiratory and subjective appetite data for one participant were excluded in experiment 2 due to fidgeting during the measurement period and was not included in analyses. A sufficient estimated sample size was based on a single group mean from our previous work [27]. The Research Ethics Board at Ryerson University approved the study and the study was registered at clinicaltrials.gov (NCT02200796). Inclusion criteria were children who were habitual breakfast consumers and able to consume the assigned foods. Participants were excluded if they were dieting, taking any medications that would affect study outcomes, had food allergies or sensitivities to test foods, or had any significant learning or behavioral difficulties. While body composition may influence DIT in adults [28], it has consistently been shown that DIT is not different between obese and normal weight children following isocaloric test meals [29,30,31]. As such, our inclusion/exclusion criteria did not include body composition.

### 2.2. Treatment Conditions

All breakfast meals were isocaloric (450 kcal), matched for 34% fat content and sodium (909 mg) and are summarized in Table 2. The control breakfast was composed of toasted Wonder™ White + *Fibre* bread (Weston Bakeries Ltd., Toronto, ON, Canada), butter (Gay Lea^®^ Foods Co-operative, Mississauga, ON, Canada), and strawberry jam (President’s Choice^®^, Brampton, ON, Canada). The egg-based protein breakfasts were served as an egg-omelet with baked home-fried potatoes (donated by McCain Canada Ltd., Florenceville-Bristol, NB, Canada), cheese and ketchup (President’s Choice^®^, Brampton, ON, Canada). Egg whites and cheese were used in varying amounts to manipulate protein content while keeping all treatments isocaloric (whey and casein; 18% in MP and HP; 16% in LP). Treatment meals were served in three equal portions, each spaced 10 min apart, providing a total of 30 min for meal completion. This provided a reasonable amount of time for children to comfortably finish their meal without feeling rushed and reduced the potential for adverse gastrointestinal symptoms (i.e., indigestion, nausea, etc.). All breakfast meals were served to participants in individual cubicles to avoid distractions from other research participants.

### 2.3. Visual Analogue Scales

In experiments 1 and 2, participants were asked to complete visual analogue scales (VAS) for motivation-to-eat and physical comfort; each VAS was a 100 mm line where they placed a pencil mark to describe their feelings along the continuum. For motivation-to-eat, children described their desire to eat (“Very weak” to “Very strong”), hunger (“Not hungry at all” to “As hungry as I have ever felt”), fullness (“Not full at all” to “Very full”), and prospective food consumption (PFC) (“A large amount” to “Nothing at all”) for subjective appetite, as reported [34,35,36,37] and validated [27] in our previous studies. A subjective appetite score was calculated from the motivation-to-eat VAS questionnaire using the following formula:

Subjective Appetite (mm) = [desire to eat + hunger + (100 − fullness) + PFC]/4.(1)

Subjective thirst was assessed via VAS using the question “How thirsty do you feel?” anchored by “Not thirsty at all” and “As thirsty as I have ever felt”. The pleasantness of the meals was assessed via VAS with the question “How pleasant did you find the breakfast/pizza?”

### 2.4. Experimental Design

Both experiments used randomized, within-subject, repeated-measures designs. Prior to participating in the study sessions, each participant attended a screening visit at Ryerson University where written consent from parents/guardians and assent from the child were obtained. Anthropometrics and body composition were measured, pizza preference for the ad libitum test meal was recorded and children were familiarized with the facility and study protocols. Each child’s height (m) and mass (kg) were measured, body mass index (kg/m^2^) was calculated and percentiles were obtained from the Centers for Disease Control and Prevention (CDC) growth charts [33]. Skinfold measurements (mm) at the tricep, bicep, suprailiac and subscapular were obtained using a Harpenden skinfold caliper (Cambridge Scientific Industries, Cambridge, MD, USA) to the nearest 0.1 mm by a trained technician. The mean of three consecutive skinfold measurements was used to determine percent body fat and fat-free mass using age- and sex-specific regression equations (Table 1) [38].

#### 2.4.1. Experiment 1

Each participant arrived at the laboratory between 0700 and 0930 h on each of their four test visits, at least one week apart, having been asked to fast for 10–12 h. Upon arrival, baseline (0 min) subjective appetite was measured via VAS [27,39], and blood glucose (BG) was measured via finger prick. Test sessions were rescheduled for participants who reported on a compliance survey not having fasted or whose BG exceeded 5.5 mmol/L. Participants then consumed, in a random order, one of four isocaloric breakfast meals (450 kcal) of varying protein content within 30 min: an egg-omelet with a side of home-fried potatoes (15 g [low protein, LP], 30 g [medium protein, MP], 45 g [high protein, HP]), or white bread with butter and jam (7 g [control, C]; Table 2). Subjective appetite was assessed via VAS immediately following breakfast consumption (30 min), and at regular intervals for a total of 4 h (60, 90, 120, 180, and 240 min). Blood glucose was measured immediately after breakfast consumption (30 min), and at regular intervals for 4 h (60, 120, 180, and 240 min). Three and a half hours from the end of breakfast consumption, participants were given an ad libitum pizza lunch with instructions to eat until they were comfortably full. Both breakfast and lunch meals were served with 500 mL bottled spring water (Nestlé Pure Life^®^, Guelph, ON, Canada). The pleasantness of the test meals was assessed using VAS [27], and pizza preferences were standardized within each participant across all sessions. Subjective physical comfort was assessed using VAS after each BG measurement.

#### 2.4.2. Experiment 2

Participants arrived at the lab between 0730 and 0800 h following a 10–12 h overnight fast and having refrained from physical exertion for 24 h. Test sessions were rescheduled if participants did not comply with the pre-test protocol. Participants rested for 30 min, followed by a measurement of resting energy expenditure (REE) for 30 min using indirect calorimetry (Parvo Medics TrueOne 2400). Participants consumed, in random order, one of two test breakfasts (HP or control) within 30 min, followed by energy expenditure measurements for 5 h. Diet-induced thermogenesis was calculated as the increase in energy expenditure above baseline REE over the 5 h. Respiratory exchange ratio (RER) was determined to assess the impact of the breakfast meals on substrate utilization. Subjective appetite was determined at baseline, immediately after breakfast consumption, and every hour for 5 h.

### 2.5. Food Intake

In experiment 1, ad libitum FI was measured from a pizza meal and FI was determined by weighing the meal before and after serving [34,35]. The pizzas were small and round (5-inch, 200 kcal) and available in two varieties with similar nutritional composition (Deep ‘N Delicious Pepperoni or Three Cheese, McCain Canada Ltd., Florenceville-Bristol, NB, Canada). Each pizza lacked an outer crust, which resulted in a more uniform distribution of energy [27,39]. The net weight of the test meal was converted to kcal based on manufacturer information. Pizza preference was determined at the screening visit. Three trays of pizza (~1800 kcal in total) with each tray containing three pizzas, two of their first choice and one of their second choice, were provided and cut into equal pieces. Participants were informed that additional hot tray replacements would be presented in 10 min intervals and to eat until comfortably full, providing a total of 30 min for meal completion. This provided a reasonable amount of time for children to comfortably finish their meal without feeling rushed and reduced the potential for adverse gastrointestinal symptoms (i.e., indigestion, nausea, etc.) Participants ate their meal in individual cubicles, which eliminated distractions from other research participants. Water intake (g) was determined by weighing each water bottle before and after the test meal.

### 2.6. Glycemic Response

In experiment 1, children’s fingers were sanitized with single-use alcohol wipes and capillary blood was collected at six time points using a single-use, auto-disabling finger-prick device. The blood sample was analyzed immediately for BG using a commercially available glucometer (Accu-Chek^®^ Aviva, Toronto, ON, Canada).

### 2.7. Diet-induced Thermogenesis

In experiment 2, participants rested in a supine position for 30 min to reach a steady resting state in an isolated, dimly lit room under controlled temperature and humidity conditions. Resting energy expenditure was determined by indirect calorimetry (ParvoMedics TrueOne2400 automated metabolic gas analysis system, ParvoMedics, Sandy, UT, USA) under a ventilated hood for 30 min. Participants were instructed to remain awake and to not move, fidget or talk while under the ventilated hood. Resting energy expenditure was calculated based on the volume of oxygen consumed (VO2) and volume of carbon dioxide (VCO2) produced using Weir’s formula [20]: REE (kcal/day) = 5.616 * VO2 (mL/min) + 1.584 * VCO2 (mL/min) [40]. Steady state was defined as a stable VO2 and respiratory exchange ratio (RER) within 10% and 5% deviation, respectively, for a minimum of 10 min. Measurements from the first 5 min were discarded to account for acclimatization to the ventilated hood system.

After REE was measured, participants consumed either a control or HP breakfast (Table 2) followed by a 10 min rest period. Respiratory gases were measured in 30 min intervals for 5 h under the ventilated hood using the indirect calorimeter, with a 30 min break between measurements. Participants could use the restroom, rest quietly, or watch TV during 30 min break periods. Diet-induced thermogenesis (kcal/h) was calculated as the increase in energy expenditure above baseline REE for 300 min. Respiratory exchange ratio was monitored to assess the impact of the test breakfast on substrate utilization. Energy expenditure and substrate oxidation rates for each hourly interval were calculated using average VO2 and VCO2 measurements [40]. Substrate oxidation was calculated using the following equations [41]:

(2) $$\mathrm{Carbohydrate}\;\mathrm{oxidation}\;={\;F}_{g\;}\times \frac{{\mathrm{VO}}_{2}}{0.746},\;\mathrm{where}\;F_{g}=\;\frac{\mathrm{RQ}-0.705}{1-0.705}$$

(3) $$\mathrm{Fat}\;\mathrm{oxidation}\;=\;F_{f\;}\times \frac{{\mathrm{VO}}_{2}}{2.03}\;\mathrm{where}\;\mathrm{Ff}\;=\;1\;-\;F_{g}$$

### 2.8. Statistical Analyses

In experiment 1, a two-way repeated measure analysis of variance (ANOVA), adjusted for multiple comparisons using a Tukey post-hoc was used to determine the effects of breakfast treatments (LP, MP, HP, control) and time on change from baseline subjective appetite and absolute BG. Change from baseline subjective appetite was used to correct for participant differences at baseline and was calculated by subtracting the baseline measurement from subsequent measurements. A one-way ANOVA using a Tukey post-hoc correction, was used to determine the effect of treatment on blood glucose incremental area under the curve (BG iAUC), FI, water intake, and pleasantness (breakfast and lunch). Blood glucose iAUC was calculated using the trapezoid method [42]. In experiment 2, a two-way repeated measures ANOVA, corrected for multiple comparison using a Tukey post-hoc, was used to assess the effect of treatment (HP, control) and time (over 5 h) on DIT (kcal/h) and change from baseline subjective appetite. Diet-induced thermogenesis (kcal), RER, carbohydrate oxidation (g/h), and fat oxidation (g/h) were assessed by paired t-test. Pearson correlations were used to determine associations between DIT and subjective appetite (experiment 2). All results are expressed as mean ± SEM. Statistical analyses were conducted using SAS version 9.3 (SAS Institute Inc., Carey, NC, USA), with significance defined as *p* < 0.05.

## 3. Results

### 3.1. Experiment 1

#### 3.1.1. Food and Water Intake

There was a main effect of breakfast meal on FI (*p* < 0.05). Food intake was lower following the HP breakfast (858 ± 88 kcal) than control (1084 ± 83 kcal, *p* = 0.005, *n* = 17) and LP (1062 ± 93 kcal, *p* = 0.01, *n* = 17) treatments (Table 3). Food intake was similar following consumption of the control, LP and MP meals. There was no significant effect of breakfast meal on water intake (*p* = 0.21).

#### 3.1.2. Subjective Ratings from Visual Analogue Scales

**Figure 1:**
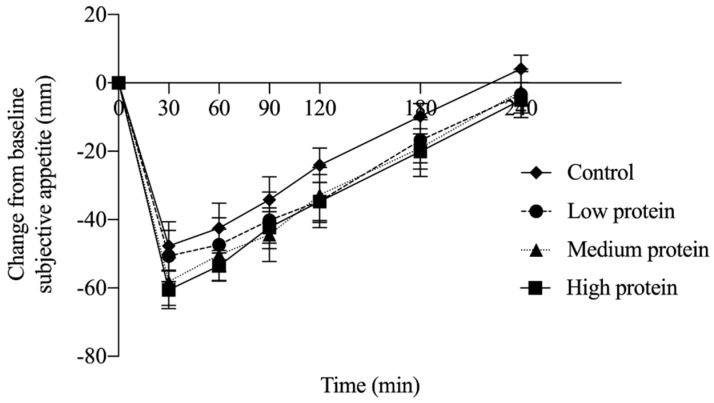
Change from baseline subjective appetite among treatments containing varying amounts of protein (experiment 1; *n* = 17). No interaction of time and treatment was observed (*p* = 1.000), however subjective appetite following the control treatment was significantly higher than LP (*p* = 0.0008), MP (*p* < 0.0001) and HP (*p* < 0.0001) treatments.

An effect of time (*p* < 0.0001) and treatment (*p* < 0.0001) were observed for change from baseline subjective appetite, but no time by treatment interaction (*p* = 1.000). Change from baseline subjective appetite after LP (*p* = 0.004), MP (*p* < 0.0001) and HP (*p* < 0.0001) were lower than the control (Figure 1; *n* = 17). Neither pleasantness of the breakfast (*p* = 0.352) nor pizza lunch (*p* = 0.319) were affected by treatment (Table 3).

#### 3.1.3. Glycemic Response

**Figure 2:**
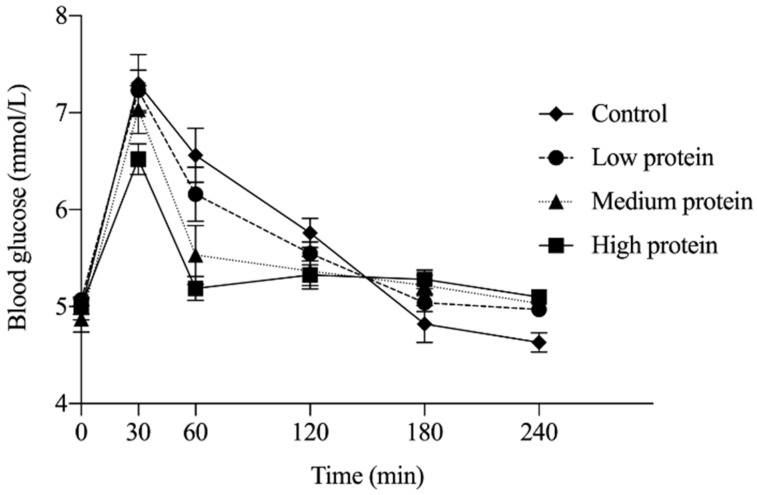
Glycemic response over 240 min following breakfast consumption; *n* = 15. At 60 min, blood glucose following consumption of the medium protein and high protein breakfast was significantly lower (MP, *p* = 0.0064; HP, *p* < 0.0001) compared with the control treatment by two-way repeated measures ANOVA with a Tukey post hoc correction. Values are expressed as mean ± SEM.

Two participants were excluded from analysis across all time points as insufficient blood was collected to quantify BG. No main effect of treatment (*p* = 0.11) on glycemic response was observed; however, main effects of time (*p* < 0.0001) and time by treatment interaction (*p* < 0.0001) were detected. Absolute BG concentration at 60 min was significantly lower following the MP (*p* = 0.006) and HP (*p* < 0.0001) breakfast treatments compared to the control breakfast (Figure 2; *n* = 15). No other significant differences were observed at other measurement time points. There was no significant effect of treatment on BG iAUC (*p* = 0.61).

### 3.2. Experiment 2

#### 3.2.1. Subjective Ratings from Visual Analogue Scales

**Figure 3:**
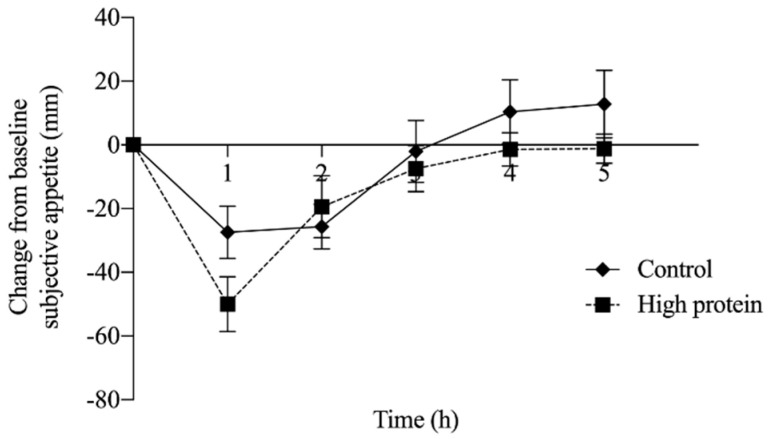
Change from baseline subjective appetite over 5 h, after consumption of the high protein or control meal (experiment 2; *n* = 9). No significant differences were observed between treatment (*p* = 0.55), time (*p* = 0.87) or time by treatment interaction (*p* = 0.99) following consumption of the high protein breakfast compared with the control breakfast.

There were no main effects of treatment (*p* = 0.55), time (*p* = 0.87), or a time by treatment interaction (*p* = 0.99) on change from baseline subjective appetite (Figure 3). Similarly, no significant association was observed between subjective appetite and DIT following consumption of either control (r = −0.3, *p* = 0.44, *n* = 9) or HP breakfast meals over the 5 h measurement period (r = −0.16, *p* = 0.69, *n* = 9).

#### 3.2.2. Diet-Induced Thermogenesis and Substrate Utilization

**Figure 4:**
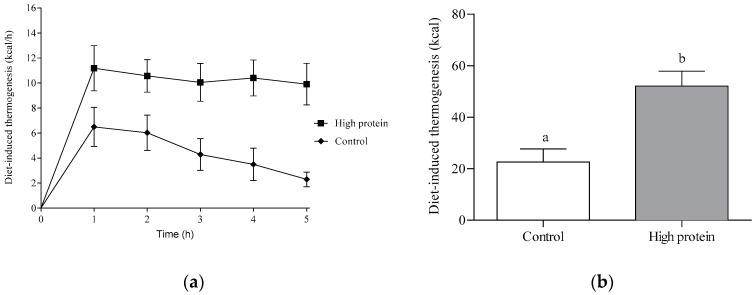
Diet-induced thermogenesis over 5 h following consumption of the HP or control meal (experiment 2; *n* = 9). (**a**) Change from baseline DIT over 5 h in children. There was a significant main effect of treatment (*p* < 0.0001) and time (*p* < 0.05), but no significant time by treatment interaction (*p* = 0.26). (**b**) Total DIT over 5 h following consumption of HP or control breakfast. DIT was significantly higher following consumption of HP (52.1 ± 5.7 kcal) compared with the control breakfast (22.6 ± 5.1 kcal; *p* < 0.0001, *n* = 9). Mean values with different letters denote a significant difference (*p* < 0.05).

Diet-induced thermogenesis (Figure 4a) was affected by both treatment (*p* < 0.0001) and time (*p* < 0.05) over the 5 h study period, but there was no significant time by treatment interaction (*p* = 0.26). Consumption of the HP breakfast resulted in greater DIT than the control treatment. Total DIT after the HP breakfast was 52.1 ± 5.7 kcal (Figure 4b), which was approximately 30 kcal higher than DIT following consumption of the control treatment (22.6 ± 5.1 kcal; *p* < 0.0001).

Furthermore, over the 5 h measurement period, there was greater fat oxidation (HP: 3.9 ± 0.2 g/h vs. C: 2.9 ± 0.2 g/h; *p* < 0.0001) and lower carbohydrate oxidation (HP: 7.6 ± 0.4 g/h vs. C: 8.6 ± 0.4 g/h; *p* = 0.04) following consumption of the HP breakfast than the control breakfast. Conversely, the control breakfast stimulated a greater rate of carbohydrate oxidation compared to the HP treatment (Table 3).

## 4. Discussion

The present study fills an important knowledge gap in our understanding of how dietary protein affects satiety and selected metabolic processes in children. Our results show that dietary protein intake affects both sides of the energy balance equation—energy intake (subjective appetite and FI) and energy expenditure (DIT). Cumulatively, these results suggest that a higher protein intake at breakfast decreases short-term FI and increases energy expenditure compared with lower protein meals.

Our findings highlight the importance of considering protein quantity when delineating the influence on satiety and FI. In experiment 1, while all test treatments suppressed subjective appetite compared to the control meal, only the HP (45 g) breakfast suppressed short-term FI compared with the LP and control treatments. Similar to the results of this study, FI following an egg-based breakfast containing 18 g of protein did not differ from a waffle-based breakfast (3 g protein) in children 8–12 y [43]. However, studies in children showed breakfast meals with greater than 30 g of protein decreased FI at lunch [23,24], suggesting that a protein intake of less than 30 g may not be enough to suppress FI.

Higher DIT was observed over 5 h following ingestion of the HP breakfast (45 g) than the isocaloric control breakfast (7 g). Studies in adults have reported that protein-rich meals (30–100 g of protein) produce larger diet-induced thermogenic effects compared to carbohydrates or fat [15,16,44]. This may reflect the lack of storage capacity for dietary protein, necessitating immediate processing [17] or higher ATP utilization for amino acids than glucose during metabolism [45]. In contrast to our results, a study in children 8–12 y found no difference in DIT following isocaloric breakfasts (340 kcal) containing 18 g and 3 g of protein [43]. This may reflect the lower protein content used or methodological differences (length of measurement period and energy content/macronutrient composition of meals) [46]. Furthermore, the type of protein may also be an important factor as high quantities (> 30 g) of complete proteins such as eggs create a surge in aminoacidemia which can drive thermogenesis [47]. Indeed, the control meal contained plant-based protein and studies suggest plant proteins may have a weaker effect on satiety than animal proteins [48,49]. Veldhorst et al. found that at 10% of energy from dietary protein there was an effect of protein source on subjective appetite, but not at 25% of energy suggesting that appetite response to protein and protein source is dependent on dose [49].

Increased postprandial glycemia is implicated in increased fat cell deposition and obesity [50]. The MP and HP breakfast treatments in our study resulted in significantly lower BG concentrations at 60 min compared to the control treatment. This result is likely due to less availability of carbohydrates in the MP and HP meals, and therefore reduced glucose response. Similarly, in adults, egg consumption at breakfast resulted in less variation of plasma glucose and insulin, and subsequently reduced energy intake over 24 h [51]. These findings may also be explained by the effect of greater quantities of protein; it has been shown that protein can attenuate glycemic response via the stimulation of insulin secretion and ultimately slowing gastric emptying [52,53,54].

Increasing dietary carbohydrate intake has been shown to increase carbohydrate oxidation and lower fat oxidation [55], while increased fat consumption may stimulate only modest increases in fat oxidation [16,29]. Indeed, fat intake exceeding the body’s ability to oxidize it results in a positive fat balance and may contribute to weight gain [56]. In the current study, the control and HP meals had differing percentages of protein (6% vs. 40%) and carbohydrates (61% vs. 27%), but fat percentage was the same (34%). Interestingly, the HP breakfast resulted in higher fat oxidation than carbohydrate oxidation, which reflects the difference in available energy sources between the two diets.

Although the current study had many strengths, there are several limitations. First, our study included a small sample size and was limited to only having short-term FI measurements. Second, while breakfast meals differing in protein content did not affect rest of day energy intake among adults [4] or adolescents [24], this has not been explored in younger children. Third, isocaloric treatment conditions were characterized by percentage of increasing protein content, however as protein content increased, carbohydrate content decreased. Therefore, while previous literature suggests the results observed in the present study can be attributed to protein content, the variation in carbohydrate content is confounding. A future study could resolve this issue by examining the effects of breakfast meals with increasing protein content with variable total energy on subjective appetite and DIT. Similarly, since all protein treatments used potatoes instead of bread as the carbohydrate source, it is more challenging to identify protein-specific effects on components of energy balance or metabolism. The white potato content of breakfast may have contributed to both appetite and FI suppression, as white potatoes have been shown to increase satiety in both adults [57] and children [58,59]. Additionally, while the largest protein meal (45 g) reduced short-term FI and increased DIT, the feasibility of parents consistently preparing meals higher in protein in free-living conditions is unknown and should be addressed in future experimental studies. Lastly, the assessment of biomarkers such as post-prandial gastrointestinal hormone concentrations or changes in plasma amino acids [54,60,61,62] would have provided valuable insight into potential physiological mechanisms of changes in subjective appetite and FI. Increasing the dietary protein content at the expense of carbohydrates in meals has a dose-dependent-like effect on satiety, glucagon-like-peptide-1 (GLP-1), peptide YY and glucagon [63]. Indeed, greater suppression of subjective ratings of hunger and ad libitum food intake 90 min after consumption of a whey protein preload (48 g) has been associated with increases in total plasma amino acids, cholecystokinin, and GLP-1 [64].

## 5. Conclusions

In conclusion, the HP diet increased satiety and DIT, and suppressed short-term FI in children 9–14 y. Future longitudinal studies are needed to assess the significance of consuming HP diets on promoting healthier body weights in children.

## Figures and Tables

**Table 1:** Participant characteristics.

Characteristic	Experiment 1 (*n* = 17)	Experiment 2 (*n* = 9)
Age (y)	12.0 ± 0.4	12.1 ± 0.5
Sex (M: F)	9: 8	6: 3
Body Mass (kg)	49.9 ± 2.7	48.9 ± 3.9
Height (cm)	154.7 ± 2.4	154.5 ± 2.2
BMI (kg/m^2^)	20.8 ± 0.9	20.4 ± 1.4
BMI Percentile	67.9 ± 5.9	64.5 ± 10.8
Fat Mass (%) ^1^	31.4 ± 2.3	27.4 ± 2.1
Fat Mass (kg) ^1^	16.2 ± 1.8	13.9 ± 2.0
Fat-Free Mass (%)	68.6 ± 2.3	72.6 ± 2.1
Fat-Free Mass (kg)	33.8 ± 1.6	35.0 ± 2.2

Data are presented as mean ± SEM ^1^. Body composition was estimated from the sum of bicep, tricep, suprailiac and subscapular regions skinfold measurements [32,33].

**Table 2:** Description of test treatments.

	Control (C)	Low Protein (LP)	Medium Protein (MP)	High Protein (HP)
Food items	white bread, butter, jam	egg yolk, egg whites, butter, cheese, home fries, ketchup	egg yolk, egg whites, butter, cheese, home fries, ketchup	egg yolk, egg whites, butter, cheese, home fries, ketchup
Energy content, kcal	450	450	450	450
Protein, g (% Energy)	7 (6)	15 (13)	30 (27)	45 (40)
Carbohydrate, g (% Energy)	69 (61)	61 (54)	45 (40)	30 (27)
Fat, g (% Energy)	17 (34)	17 (34)	17 (34)	17 (34)
Sodium, mg	909	909	909	909
Fibre, g	3	5	3	2

**Table 3:** The effect of test meals on food and water intake, and subjective pleasantness (experiment 1), and diet-induced thermogenesis and substrate utilization (experiment 2).

	Control (C)	Low Protein (LP)	Medium Protein (MP)	High Protein (HP)
**Experiment 1**				
Food intake (kcal)	1084 ± 83 ^a^	1062 ± 93 ^a^	1001 ± 85 ^a^^,^^b^	858 ± 88 ^b^
Water intake (g)	470 ± 56	445 ± 71	464 ± 62	407 ± 56
Pleasantness of breakfast (mm)	72 ± 8	84 ± 4	80 ± 6	74 ± 5
Pleasantness of pizza lunch (mm)	83 ± 6	89 ± 3	89 ± 4	91 ± 3
**Experiment 2**				
Diet-induced thermogenesis (kcal)	22.6 ± 5.1 ^a^			52.1 ± 5.7 ^b^
Mean respiratory exchange ratio	0.86 ± 0.01			0.85 ± 0.01
Mean carbohydrate oxidation (g/h)	8.6 ± 0.4 ^a^			7.6 ± 0.4 ^b^
Mean fat oxidation (g/h)	2.9 ± 0.2 ^a^			3.9 ± 0.2 ^b^

Experiment 1: *n* = 17, Experiment 2: *n* = 9; values are expressed as mean ± SEM. Mean values with different letters denote a significant difference (*p* < 0.05) using a one-way ANOVA with a Tukey post hoc correction (experiment 1) and paired t-tests (experiment 2).
